# Dataset of coral reefs monitoring, Puerto Morelos, Mexico, 2019

**DOI:** 10.1016/j.dib.2022.108253

**Published:** 2022-05-11

**Authors:** Hansel Caballero‐Aragón, Susana Perera‐Valderrama, Sergio Cerdeira‐Estrada, Raúl Martell‐Dubois, Laura Rosique‐de la Cruz, Lorenzo Álvarez‐Filip, Esmeralda Pérez‐Cervantes, Nuria Estrada‐Saldivar, Rainer Ressl

**Affiliations:** a National Commission for the Knowledge and Use of Biodiversity (CONABIO), Liga Periférico— Insurgentes Sur 4903, Parques del Pedregal, Tlalpan 14010, Mexico City, Mexico; b Biodiversity and Reef Conservation Laboratory, Academic Unit of Reef Systems, Institute of Marine Sciences and Limnology, National Autonomous University of Mexico, Puerto Morelos 77580, Quintana Roo, Mexico

**Keywords:** Coral reefs, Biological indicators, Benthos and fish communities, Mexico

## Abstract

Noticeable within the Mexican Caribbean is the Arrecife de Puerto Morelos National Park (APMNP), a marine protected area established as an essential component for managing and protecting coral reefs. In June 2019, we conducted a survey in eight shallow reef sites of the APMNP with the purpose of applying a coral reef assessment method, based on biological indicators of the condition of both benthos and fish communities. In this paper we present tables with data of biological and ecological variables such as: benthos coverage, species composition and abundance of corals, abundance of urchins and coral recruits, bleaching, coral diseases and coral mortality percent, reef relief, and composition and abundance of key commercial and herbivorous fish species. The research article related to these databases was published in the journal *Diversity* with the title: Puerto Morelos coral reefs, current state and their classification by a scoring system.

## Specifications Table

**Table:** 

Subject	Marine Biology
Specific subject area	Marine ecology. Condition assessment of coral reef benthic and fish communities.
Type of data	Tables
How data were acquired	Underwater sampling via scuba diving
Data format	Raw
Parameters for data collection	Data collection took place in the back‐reef zone, at depths from 2 to 5 m.
Description of data collection samplings	Data were collected *in situ* through:10 m‐long point‐intercept transect for benthos, 10 m-long lineal transect for corals, 10 m x 1 band transect for sea urchins, 25 cm quadrat frame for coral recruit, and a 30 × 2 m linear track for fish. The rugosity of the sites was assessed using the chain (3 m-long) methodology
Data source location	Data was gathered in eight sites of the Arrecife de Puerto Morelos National Park, located at the northeast portion of Quintana Roo state, Yucatán Peninsula, Mexico.
Data accessibility	https://doi.org/10.6084/m9.figshare.19385762.v1
Related research article	H. Caballero-Aragón, S. Perera-Valderrama, S. Cerdeira-Estrada, R. Martell-Dubois, L. Rosique-de la Cruz, L. Álvarez-Filip, E. Pérez-Cervantes, N. Estrada-Saldívar, R. Ressl, Puerto Morelos Coral Reefs, Their Current State and Classification by a Scoring System, Diversity 12 (2020) https://doi.org/10.3390/d12070272

## Value of the Data

• The data set can be incorporated into national or regional assessments (Mesoamerican Reef System) to contribute to conservation programs and management of coral reefs in marine protected areas.
• The dataset can help inform the Warning System on the Ecological Condition of Coastal Marine Ecosystems of the Marine-Coastal Information and Analysis System (SIMAR) [1].

## 1 Data Description

A total of eight Excel tables were created, corresponding to each reef site:

• DB PM Limones 2019: Data base from Limones site, with seven spreadsheets: Location, Benthos, Coral, Sea urchins, Rugosity, Coral and Fishes.
• DB PM Bonanza 2019: Data base from Bonanza site, with seven spreadsheets: Location, Benthos, Coral, Sea urchins, Rugosity, Coral and Fishes.
• DB PM Tanchacté Norte 2019: Data base from Tanchacté Norte site, with seven spreadsheets: Location, Benthos, Coral, Sea urchins, Rugosity, Coral and Fishes.
• DB PM Tanchacté Sur 2019: Data base from Tanchacté Sur site, with seven spreadsheets: Location, Benthos, Coral, Sea urchins, Rugosity, Coral and Fishes.
• DB PM La Bocana 2019: Data base from La Bocana site, with seven spreadsheets: Location, Benthos, Coral, Sea urchins, Rugosity, Coral and Fishes.
• DB PM Radio Pirata 2019: Data base from Radio Pirata site, with seven spreadsheets: Location, Benthos, Coral, Sea urchins, Rugosity, Coral and Fishes.
• DB PM Jardines 2019: Data base from Jardines site, with seven spreadsheets: Location, Benthos, Coral, Sea urchins, Rugosity, Coral and Fishes.
• DB PM La Pared 2019: Data base from La Pared site, with seven spreadsheets: Location, Benthos, Coral, Sea urchins, Rugosity, Coral and Fishes.

Each excel has seven spreadsheets with field information taken at each site. The Location sheet provides the site information. The Benthos sheet shows the percentages of substrate coverage by different groups for each sampling unit. The Coral sheet shows the identified species and other descriptive variables such as size and health condition of each colony counted. The Sea urchins sheet shows the identified species and their abundance per sampling unit. In the Rugosity sheet we present length values of the chain (3 m-long) adjusted to the contour of the reef. The Coral recruits sheet presents the species and abundance of recruits. Finally, the Fishes sheet shows the species and number of individuals of the key herbivorous and carnivorous fish species quantified by each sampling unit.

## 2 Experimental Design, Materials and Methods

### 2.1 Sampling area

**Fig. 1:**
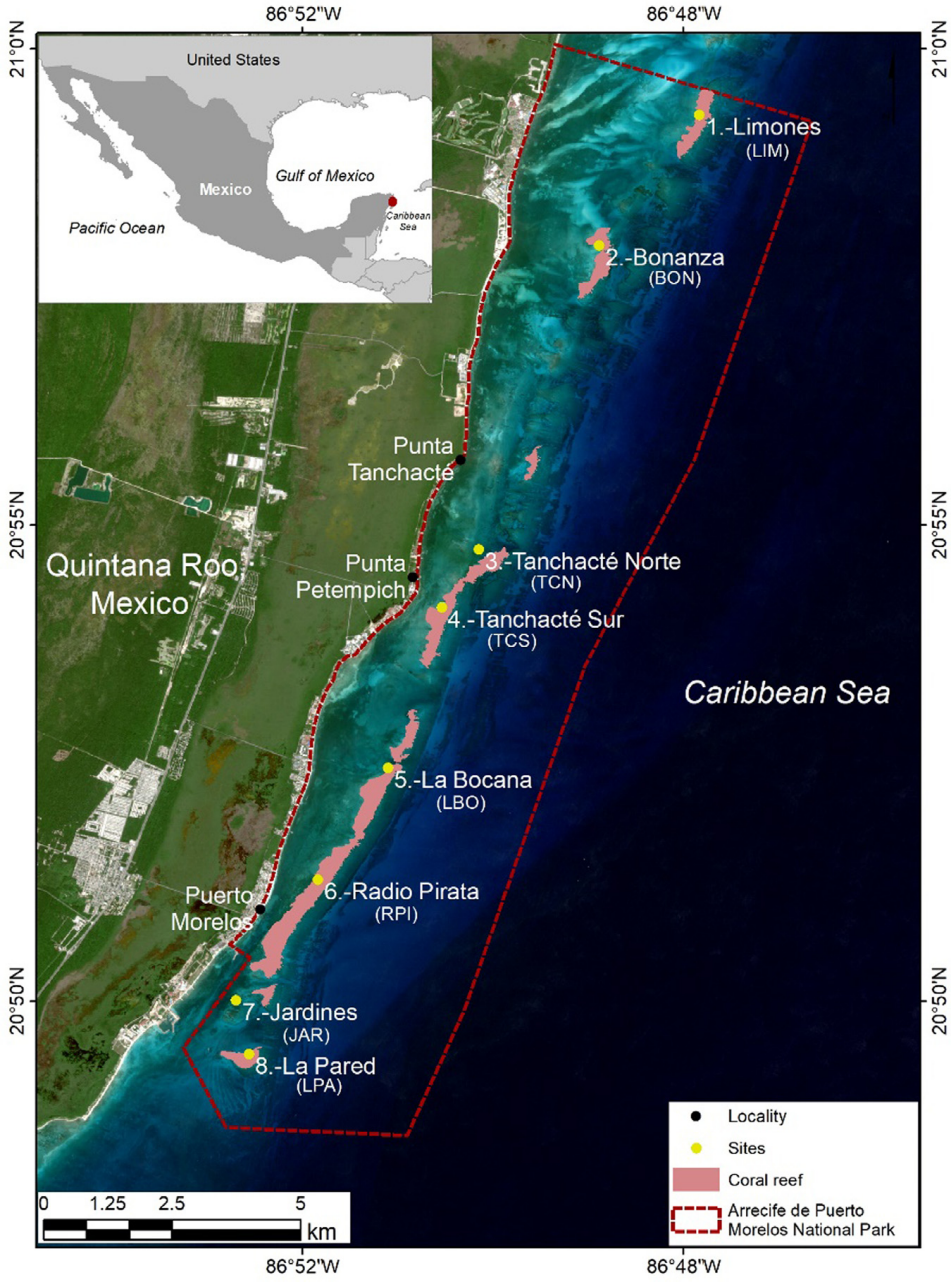
Map of the geographical location of the sampling sites within the APMNP. The acronyms of each site appear in parentheses.

The APMNP is located at the northeast portion of Quintana Roo state, Yucatán Peninsula, Mexico. The reef extends parallel to the shore, with crests segments, a reef lagoon covered by seagrass beds, and adjacent mangroves. The sampling took place at eight sites located in the back-reef zone, at depths from 2 to 5 m (Fig. 1). The reefs sampled are fixed sites of the monitoring carried out by the managers of the APMNP.

### 2.2 Sampling methodology

To obtain community variables, we used the coral reef methodology of the Monitoring Protocols for the Marine Biodiversity in Protected Naturals Areas of the Mexican Caribbean [3]. Data corresponds to biological indicators related to corals (cover percentage, abundance and condition (mortality and diseases)), algae (% cover percentage), sea urchins (abundance), fish species (abundance and size) and other components of the benthos (cover percentage).

At each sampling site, at least 10 point-intercept transects of 10 m long were located to determine the bottom cover (%), as established in the AGRRA protocol, version 2016-18 [4]. In each transect, the type of substrate was recorded every 10 cm according to 12 categories: living coral (for scleractinian corals and hydrocorals), recent mortality coral, old mortality coral, fleshy algae (leafy, filamentous, globose and corticated macroalgae), crustose coralline algae (incrusted red algae), calcareous algae (articulated), turf (cespitose algae with appreciable thickness), cyanobacteria, rubble, sand, octocorals, aggressive invertebrates (encrusting sponges and other bioeroder invertebrates), “others”. The percentage of the bottom cover by category corresponded to the total sum of points of each one of them.

Regarding the corals, all colonies ≥ 5 cm located below the transect line were evaluated. The following information was obtained from each of them: coral species, maximum diameter (cm) and perpendicular diameter (cm) (from the top view), height (cm), percent of the colony surface with old mortality (any non-living part of a coral in which the corallite structures disappeared or were covered by any other organism) and / or recent mortality (any non-living part of a coral where the corallite structures were still visible and were not covered by any other organism), signs of coral disease and bleaching (pale, partial or total bleaching).

For the assessment of the coral recruits, quadrat frames of 25 cm were located in the immediate vicinity of the 10 m transects (more than 30 units per site). The number of coral recruit colonies within each quadrat was quantified, identifying each one up to genus level. All sea urchin's individuals observed within one meter of width along the transect line were also identified and counted.

To evaluate the structural complexity of the reef, in the same sample area the chain transect method was used. For this we calculate the rugosity index (m) as the length occupied by the chain following the contour of the reef divided by the length of the stretched chain (3 m).

Key commercial and herbivorous fish [2] were sampled using a 30 m × 2 m band transect as the sampling unit. Six transects were located per site. The families Scaridae and Acanthuridae were considered as key herbivorous fishes. The families Serranidae and Lutjanidae were considered as key commercial fish. Each individual of these species observed within the sampling area was quantified and its total length was estimated using length classes (cm) in ranges from 0-5, 5-10, 10-20, 20-30, 30-40 and > 40.

## Ethical Statement

1) This dataset is the authors' own original work, and has been previously deposited in the figshare repository.
2) The paper is not currently being considered for publication elsewhere.
3) The paper reflects the authors' own work in a truthful and complete manner.
4) The paper properly credits the meaningful contributions of co-authors and co-researchers.
5) All sources used are properly disclosed with the correct citation.
6) All authors have been personally and actively involved in substantial work leading to the paper, and will take public responsibility for its content.

## CRediT Author Statement

**Hansel Caballero‐Aragón:** Conceptualization, Methodology, Software, Writing – original draft preparation, Data curation; **Susana Perera‐Valderrama:** Conceptualization, Methodology, Software, Writing – review & editing, Data curation; **Sergio Cerdeira‐Estrada:** Conceptualization, Methodology, Writing – review & editing, Supervision; **Raúl Martell‐Dubois:** Conceptualization, Methodology, Writing – review & editing; **Laura Rosique‐de la Cruz:** Conceptualization, Methodology, Writing – review & editing; **Lorenzo Álvarez‐Filip:** Conceptualization, Methodology, Writing – Review & editing; **Esmeralda Pérez‐Cervantes:** Writing – review & editing, Data curation, **Nuria Estrada‐Saldivar:** Writing – review & editing, Data curation; **Rainer Ressl:** Reviewing and Editing, Supervision.

## Declaration of Competing Interest

The authors declare that they have no known competing financial interests or personal relationships which have or could be perceived to have influenced the work reported in this article.

## Data Availability

Dataset of coral reefs monitoring, Puerto Morelos, Mexico, 2019 (Original data) (Figshare).

## Appendix. Supplementary Materials

• DB PM Limones 2019.
• DB PM Bonanza 2019.
• DB PM Tanchacté Norte 2019.
• DB PM Tanchacté Sur 2019.
• DB PM La Bocana 2019.
• DB PM Radio Pirata 2019.
• DB PM Jardines 2019.
• DB PM La Pared 2019.
